# LUNAS: A Rapid and Sensitive Nucleic Acid Detection Assay Using Split NanoLuc Luciferase Complementation

**DOI:** 10.1021/acscentsci.3c00366

**Published:** 2023-04-07

**Authors:** Longwei Liu, Yingxiao Wang, Danielle L. Schmitt

**Affiliations:** † Alfred E. Mann Department of Biomedical Engineering, University of Southern California, Los Angeles 90089-1112, California, United States; ‡ Department of Chemistry and Biochemistry, University of California Los Angeles, Los Angeles, California 90095-1569, United States; § Institute for Quantitative and Computational Biosciences, University of California Los Angeles, Los Angeles, California 90095, United States; ∥ Molecular Biology Institute, University of California Los Angeles, Los Angeles, California 90095, United States

**Figure 1:**
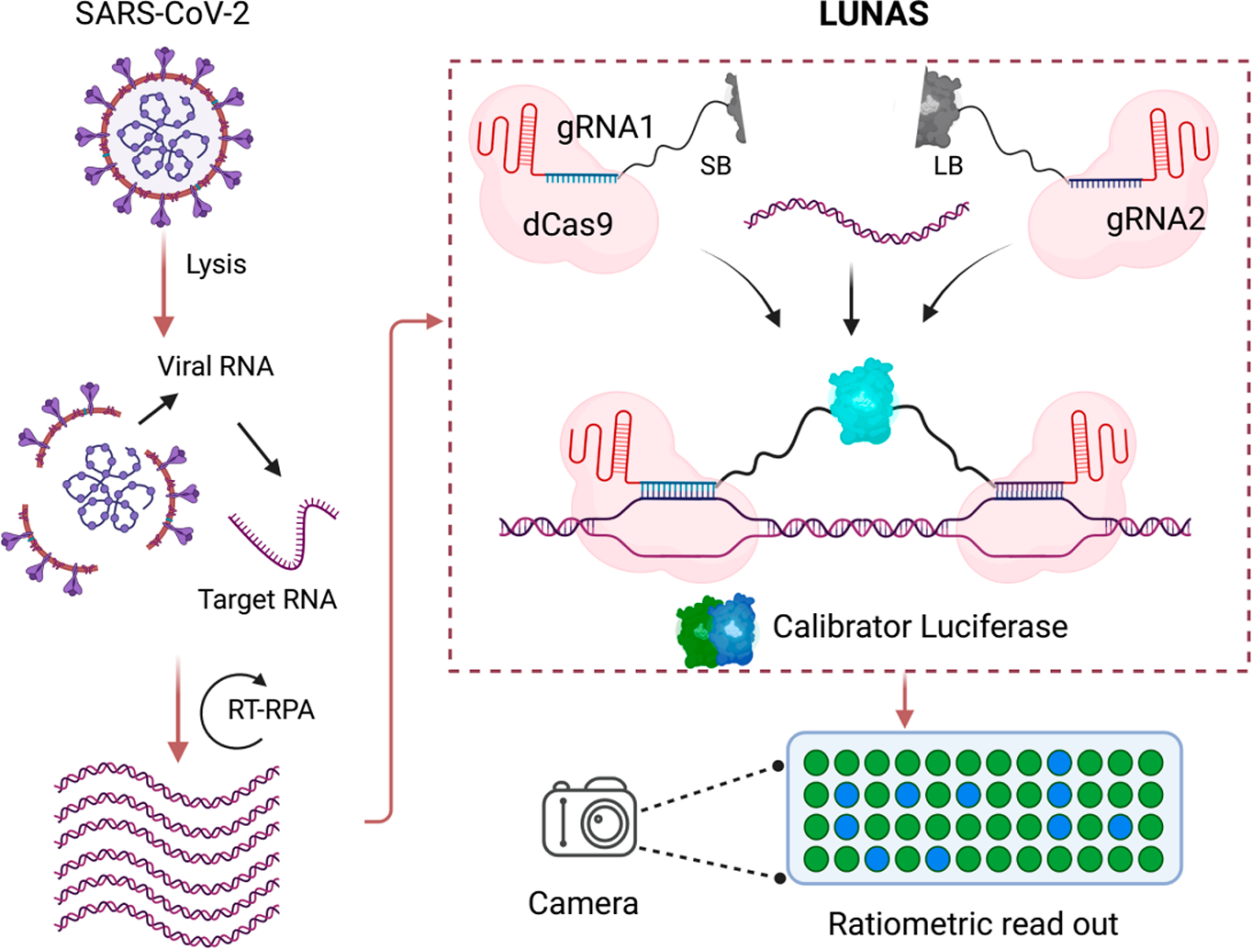
Schematic overview of the (RT)-RPA-LUNAS method. (RT)-RPA-LUNAS LUNAS employs a combination of recombinase polymerase amplification (RPA) and split NanoLuc luciferase complementation to detect the presence of target nucleic acid in a sample with high sensitivity. SB: small BiT; LB, large BiT.

Detection of viral nucleic acids using rapid, portable, and user-friendly methods at point-of-care is of extreme importance during disease outbreaks, especially those of emerging viral infections. The ability to quickly detect and diagnose viral infections is essential for disease mitigation. Although current approaches for detecting viral nucleic acids are sensitive and effective, they often require expensive equipment and highly trained personnel. In this issue of *ACS Central Science*, Merkx and co-workers present an adaptable, bioluminescence-based point-of-care diagnostic technique capable of sensitively and robustly detecting viral nucleic acids^1^ (Figure 1).

**Figure 2:**
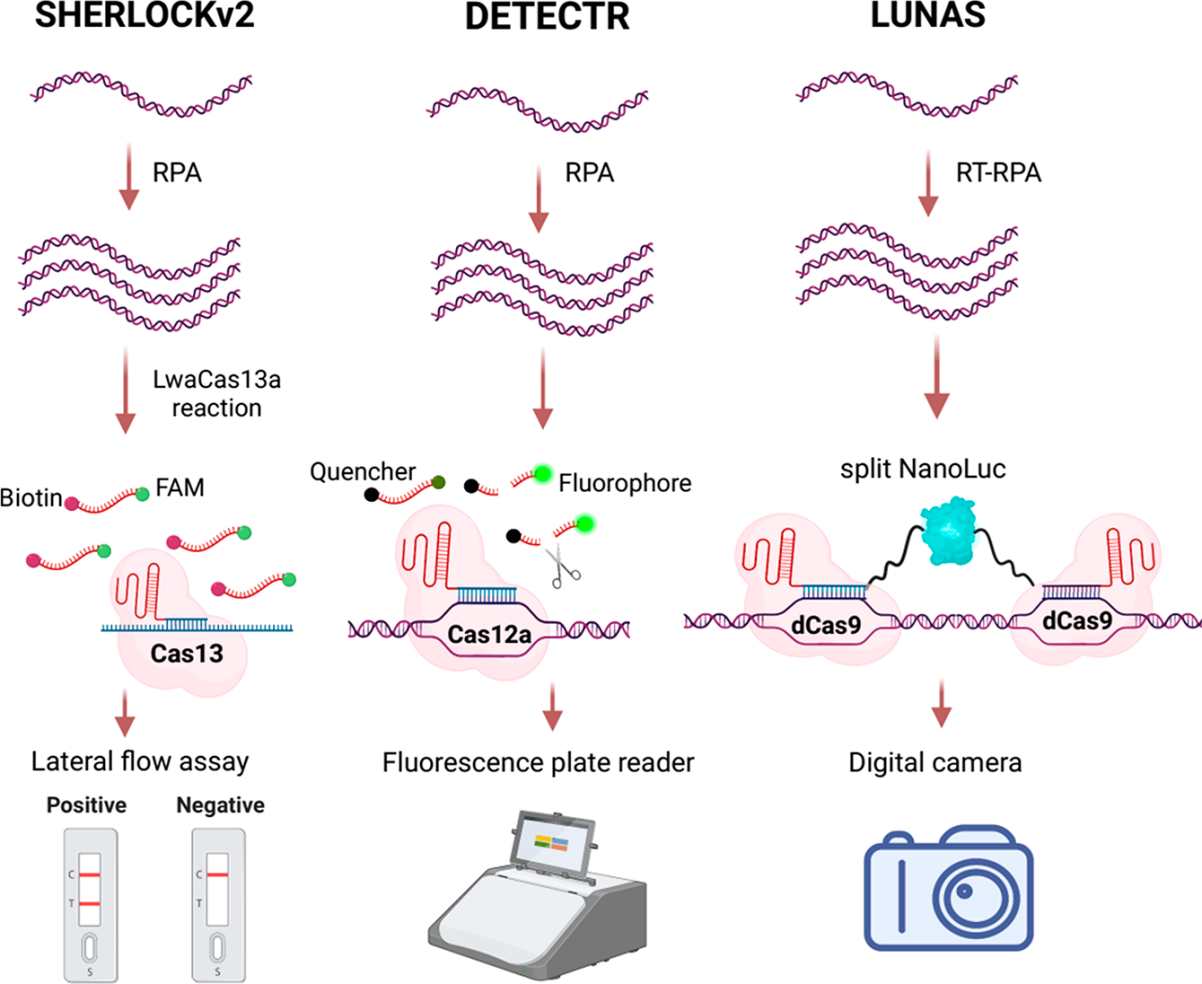
Examples of CRISPR-Cas technologies for nucleic acid detection. (Left) SHERLOCKv2 is an updated version of the SHERLOCK (Specific High-sensitivity Enzymatic Reporter unLOCKing) nucleic acid detection platform that has been engineered to provide a visual colorimetric readout using commercial lateral flow strips. Briefly, viral nucleic acid is amplified using RPA, and a Cas13a derived from *Leptotrichia wadei* (LwaCas13a) is used to bind to the nucleic acid of interest and engage in collateral cleavage of nearby nontargeted reporter RNAs. Reporter RNAs contain both a biotin label and fluorescein (FAM) label for lateral flow assays. (Middle) In the DETECTR (DNA endonuclease-targeted CRISPR trans reporter) method, RPA is also used to amplify viral nucleic acid. Cas12a-dependent recognition of pathogenic nucleic acids turns on collateral nuclease activity that cleaves DNA probes, relieving fluorescence quenching on the reporter probe, resulting in a stable and strong fluorescent signal. (Right) LUNAS employs dCas9 and a split NanoLuc luciferase complementation to detect the presence of target nucleic acid in a sample.

The most common technique used for detection of viral nucleic acids is polymerase chain reaction (PCR). However, PCR requires equipment and personnel that might be unavailable in the field, resulting in long lag-times between sample collection and results. Recently, many groups have explored other methods for detecting viral nucleic acids, particularly using CRISPR-based methods for sequence-specific detection. For example, previous CRISPR-based approaches use Cas12 or Cas13, RNA-guided endonucleases, to specifically bind to viral nucleic acids. Cas-mediated cleavage of a nucleic acid-based probe is used to release a fluorescent or colorimetric signal as a diagnostic readout for several viruses, including human papillomavirus (HPV), Zika, and SARS-CoV-2.^2−7^ However, these approaches require an hour or more for detection, and their readout requires a plate reader or nonquantitative laminar flow assay (Figure 2).

To develop more rapid and easy-to-deploy point-of-care diagnostics, Merkx and co-workers turned to bioluminescence. Bioluminescence requires no external excitation sources, only a luciferase substrate. As Merkx et al. show, bioluminescent signals can be quantitatively detected using a commercial point-and-click camera. Inspired by a previous dCas9-based split firefly luciferase complementation sensor for double stranded DNA,^8^ which uses an inactive version of the nuclease Cas9 that can bind to but not cleave DNA, Merkx et al. present Luminescent Nucleic Acid Sensor (LUNAS) to detect viral nucleic acid. LUNAS consists of dCas9 fused to either the small BiT or large BiT fragments of split NanoLuc luciferase preloaded with one of two short guide RNAs specific for adjacent regions of viral nucleic acid. NanoLuc luciferase was chosen as it is brighter, smaller, and more stable than firefly luciferase. As such, in one pot, the loaded dCas9, a calibrator luciferase, luciferase substrate, and recombinase polymerase amplification (RPA) reaction are mixed (RPA-LUNAS). Viral nucleic acid is amplified via RPA, and each dCas9 binds to the amplified nucleic acid. The short distance between guide RNA complementation sites enables reconstitution of full-length NanoLuc and production of bioluminescence within 30 min. A calibration luciferase fused to the fluorescent protein mNeonGreen, which undergoes bioluminescent resonance energy transfer (BRET) with mNeonGreen, is included to correct for luciferase substrate depletion and produce a quantitative ratiometric readout, simplifying results. While LUNAS can be used without RPA for detection of viral nucleic acid to ∼1 pM, amplification allowed for detection of viral nucleic acid down to 200 copies of target nucleic acid, or attomolar sensitivity. To demonstrate the feasibility for point-of-care diagnostics, RPA-LUNAS was used with a commercial point-and-click camera to detect SARS-CoV-2 from clinical nasopharyngeal samples (Figure 1). RPA-LUNAS was able to sensitively and specifically detect SARS-CoV-2 in patient samples, with results similar to those obtained using PCR-based methods.

LUNAS is capable of sensitively detecting SARS-CoV-2 in patient samples, with relatively good accuracy. LUNAS is a highly modular technique, with potentially broad application. For instance, since LUNAS uses dCas9-guide RNA complexes to recognize and bind to complementary nucleic acids, guide RNA could be developed for nucleic acids from other viruses, broadening the applicability of this approach. The modularity of the reporting unit means multiplexing of LUNAS with dCas9 fused to other reporters could be used to detect multiple infections at once, furthering the application of LUNAS for point-of-care diagnostics. An exciting application of LUNAS would be for *in vivo* diagnosis. To achieve this, some complexity of LUNAS would need to be overcome, with a focus on developing an entirely genetically encoded system or engineering delivery for sensitive, *in vivo* detection of viral nucleic acids. Whereas this work focused on viral nucleic acids, other related techniques have been used for genotyping,^2^ which could be an exciting cell-based or *in vivo* application for LUNAS. Ultimately, the development of LUNAS by Merkx and co-workers represents a step forward in the development of rapid, user-friendly point-of-care diagnostics that can be easily adapted for the next disease outbreak..
